# Classification of speech nasality of individuals with cleft lip and palate with distinct ordinal scales

**DOI:** 10.1590/2317-1782/e20240044en

**Published:** 2025-01-27

**Authors:** Gisele Fonseca do Carmo, Jeniffer de Cássia Rillo Dutka, Flora Taube Manicardi, Beatriz Campanine Geremias, Maria Inês Pegoraro-Krook, Viviane Cristina de Castro Marino

**Affiliations:** 1 Programa de Pós-Graduação em Fonoaudiologia, Universidade Estadual Paulista “Júlio de Mesquita Filho” – UNESP - Marília (SP), Brasil.; 2 Pós-Graduação em Ciência da Reabilitação, Hospital de Reabilitação de Anomalias Craniofaciais, Universidade de São Paulo – USP - Bauru (SP), Brasil

**Keywords:** Cleft Palate, Velopharyngeal Insufficiency, Speech Disorders, Speech Perception, Speech

## Abstract

**Purpose:**

To investigate whether there is a difference in the classification of speech hypernasality by inexperienced listeners using different ordinal scales; to verify the agreement of the listeners in the analyses when using these scales; and to verify whether the order in which the scales are presented influences the results.

**Methods:**

Twenty Speech-Language Pathology students classified the degrees of hypernasality of 40 (oral) samples from patients with cleft lip and palate. Ten performed the classifications using a 4-point scale (absent, mild, moderate, and severe) and, after two weeks, using a 3-point scale (absent, slightly hypernasal, and very hypernasal). Other ten students performed the same classifications, but in reverse order. The classifications were made remotely and documented on a form.

**Results:**

The average percentage of correct responses by the students, in relation to the gold standard, was significantly higher for the 3-point scale. There was no significant interaction between the order of presentation and the scale for the percentage of correct classifications. The students' agreement with the gold standard assessment was fair (3-point scale) and moderate (4-point scale). The mean percentage of agreement of the intra-rater analyses was significantly higher for the 3-point scale. There was no significant interaction between presentation order and scale for the percentage of intra-rater classifications. The Kappa coefficient index showed more favorable intra-rater agreement for the reduced scale.

**Conclusion:**

The reduced scale favored the classification of speech hypernasality by listeners and can be considered an important strategy to favor the initial evaluations of students in Speech Therapy during their training.

## INTRODUCTION

Hypernasality is an important speech symptom in the presence of velopharyngeal dysfunction (VPD) after surgical correction of the palate^(1,2)^. Auditory-perceptual assessment is essential to identify this speech symptom^(3)^. Through this assessment, clinicians can identify and measure the severity of hypernasality^(4)^, which facilitates clinical decision-making^(3)^, in addition to allowing the monitoring of the results achieved^(5)^. However, this assessment is based on the listener's auditory impressions^(5)^ and is susceptible to errors and biases, even when performed by experienced professionals^(1,3,6)^.

Reliably assessing speech characteristics related to VPD is a challenging process, since variations in the results of perceptual assessments of hypernasality can be justified by the degree of clinical experience and the criteria adopted by each evaluator in their analyses^(5)^. Other variables that may influence the auditory-perceptual assessment, affecting its reliability, include the type, extent and phonetic context of the speech stimulus^(7-9)^ and also the presence of coexisting alterations^(10)^.

Several strategies are recommended to reduce the biases of subjectivity present in this assessment method, with emphasis on the establishment of scoring criteria (through the use of scales) for the classification of hypernasality^(5,8)^. The use of appropriate scales emerges as a crucial strategy to improve the reliability of the classification of hypernasality^(1,3,8,11)^. Different types of scales are described in the literature, ranging from equal or ordinal interval scales, direct magnitude estimation, visual analogue scale^(5)^ to the Borg scale^(8,12)^.

The debate surrounding the types of scales and methods that can be used to improve auditory-perceptual assessment continues among researchers^(5).^ Proportion scales, especially the Borg scale, offer the perspective of increasing the reliability of hypernasality ratings by different evaluators^(8,12)^. However, these scales have disadvantages for clinical applications. The direct estimation scale, for example, is considered impractical for routine use in clinical settings because it requires more extensive training of evaluators^(13)^. In contrast, scales with equal intervals are the most frequently used in auditory-perceptual assessment^(6,9)^ and are considered the most appropriate for use in a clinical context because they allow evaluators to obtain ratings relatively easily^(14)^, allowing comparison of results between scales and evaluators.

In the interval scale, the evaluator assigns an index (or category) to the speech aspect assessed, indicating its level of severity. The lowest value (category) refers to the absence of alteration, while the highest value (category) indicates the maximum degree of alteration^(15)^. The literature indicates variability regarding the score to be used by categorical scales to classify hypernasality, which may vary in three points^(16-18)^, four points^(19,20)^, five points^(4,12)^ or even more points^(21)^. The four-point scale is frequently used, following the recommendations of the universal parameters for speech documentation in cleft lip and palate (CLP)^(11)^.

In an attempt to minimize the difficulties associated with the task of classifying speech hypernasality by inexperienced listeners, such as children, some researchers^(16)^ have proposed the use of a three-point scale (normal, slightly hypernasal, and very hypernasal) instead of a scale with a greater number of options. They argued that the use of scales with more options may result in less reliable assessments among inexperienced listeners due to possible adverse interaction with the scale itself. In a previous study^(16)^, the results showed that the hypernasality classifications made by children were compatible with those made by the experienced professional. According to the authors, the justification for these findings is related to the use of the reduced scale, which, by offering fewer options, facilitated the children's responses. Two other studies also used three-point ordinal scales to classify hypernasality, however, the classifications were made by experienced speech-language pathologists^(17,18)^. It is therefore observed that only three studies used reduced categorical scales (three points) to classify speech hypernasality, and only one of them involved listeners without experience in the analyses. Given the subjective nature of the auditory-perceptual assessment and considering that scales with a higher number of points (e.g., four points) can complicate the classification of hypernasality by inexperienced listeners, the question arises as to whether Speech-Language Pathology students could benefit from using a reduced scale, as proposed in a previous study^(16)^. Thus, the objectives of the study were (1) to investigate whether there is a difference in the classification of speech hypernasality of individuals with CLP by inexperienced listeners (Speech-Language Pathology students) using different ordinal scales (3 and 4 points), (2) to verify the agreement of the listeners in the analyses when using these two scales, and (3) to verify whether the order in which the scales are presented influences the results.

## METHODS

The study was approved by the Human Research Ethics Committee of the place where it was conducted (doc N. 5.679.783). All participants who agreed to participate in the study signed the Free and Informed Consent Form. Pre-existing speech recordings of individuals with a history of CLP, of both sexes, were used. These recordings were subsequently evaluated by 20 participants, using different interval scales.

### Speech samples

The speech recordings were selected from a pre-existing set in a database and were originally obtained directly from the computer, equipped with a *Sound Blaster Audigy 2* sound card and *Sony® Sound Forge* software, version 8.0, with a sampling rate of 44100 Hz, in single channel, 16 Bits. The audio signal was captured using a head- microphone (model AKG C420®), in an acoustically treated room. All recordings in the study had good audio quality, with a standardized interval of one second between each sentence. The speech stimulus comprised a set of 12 sentences consisting predominantly of high intraoral pressure phonemes, in which each sentence was composed of a single target sound in recurrence, following the methodology of previous studies^(9,22)^.

The study included 40 speech samples (in audio) from individuals with CLP. The speech samples were selected from a database of recordings previously grouped according to the degree of nasality. The first author of the study listened to the recordings and the first 10 speech samples with good recording quality that were considered (by the author) to be representative of each of the four degrees of nasality (A = absent hypernasality, HL = mild hypernasality, HM = moderate hypernasality and HG = severe hypernasality) were selected for the study. The selected speech samples did not present dysphonia. Other speech symptoms, such as nasal air emission/turbulence, compensatory articulation, were not controlled in the present study.

### Gold-standard perceptual assessment

The recordings selected for the study were analyzed by experienced speech therapists at two different times. The degree of hypernasality on the 4-point scale was pre-established, as described in a previous study^(22)^ and enabled the creation of a database in which the first author selected the 40 recordings included in the study, 10 (25%) consisting of speech samples representative of HA, 10 (25%) consisting of speech samples representative of HL, 10 (25%) consisting of speech samples representative of HM and 10 (25%) consisting of speech samples representative of HG. The first author, when selecting the 40 speech samples, agreed with the gold-standard assessment reported in a previous study^(22)^.

The 3-point scale, however, had not yet been applied to the 40 selected recordings. The procedure for analyzing the recordings, applying the 3-point scale (A = absent hypernasality, PH = little hypernasality and MH, a lot of hypernasality) was performed for the present study by experienced speech therapists, and the same 40 previously selected recordings were regrouped, with 23 (30%) consisting of samples representative of A, 9 (22.5%) of samples representative of PH and 19 (47.5%) of samples representative of MH. When regrouping the recordings, there was agreement in the analyses of at least two of the three experienced speech therapists, with no disagreements among them. All speech therapists who performed analyses of speech samples included in the study routinely participate in speech evaluations of patients with CLP and work in the same center for the management of craniofacial anomalies. The analyses of the speech samples performed by the experts, using each of the scales, were adopted as the gold-standard criterion for comparisons with the participants' assessments.

### Participants

Twenty students, aged between 20 and 40 years of age (mean age 21 years and 3 months), from an undergraduate course in Speech-Language Pathology and Audiology and native speakers of Brazilian Portuguese, participated in this study. All students were enrolled in the second year of their undergraduate course and had not yet taken the theoretical course in the area of ​​CLP. Furthermore, all participants reported having normal hearing and no previous experience in the speech assessment of individuals with CLP.

### Procedures

The 20 study participants were divided into two groups: Group 1 (G1) and Group 2 (G2), each composed of 10 students, randomly selected to conduct the analysis of the speech samples using the two proposed scales. All participants in Groups G1 and G2 performed the classification of 40 speech samples, including 20 duplicate samples for a subsequent analysis of intra-rater agreement, totaling 60 speech samples analyzed using each of the scales. The presentation of all samples was conducted randomly.

Students in Groups G1 and G2 conducted the analysis of the samples individually through an online meeting, using their own headphones. The analyses were performed in two distinct stages. In Stage 1, participants in G1 classified the 40 speech samples (plus 20 repetition samples) using a 4-point scale (A, HL, HM, HG), while participants in G2 classified the same 40 speech samples (plus 20 repetition samples) using a 3-point scale (A, PH, MH). In Stage 2, participants in G1 reclassified the same 40 speech samples (plus 20 repetition samples) using a 3-point scale (A, PH, MH). On the other hand, participants in G2 performed this task, but using a 4-point scale (A, HL, HM, HG). There was a 2-week interval between the two stages of the study.

At each stage of the study, the speech samples were presented by the first author (G. F. C.) via the Google Meet platform to one or more students who made up each group. In total, each student participated in two meetings, with an average duration of 70 minutes per meeting. The duration of each session included the time dedicated for the student or group of students to join the videoconference; the PowerPoint presentation followed by the researcher's technical instructions; the period intended to clarify possible doubts; and also the pre-established pause time during the collection.

A PowerPoint presentation was made available to the students in each group before the activities began. This presentation included concise definitions of CLP, VDF, and hypernasality, with the aim of familiarizing the students with the topic. No reference samples or information that could influence their analyses were included. The students were informed that each speech sample would be presented once, with the option of repeating it if necessary. They were also instructed to use the same headset and be in the same quiet environment during both stages of the study.

In each stage of the study, the students listened to the 60 speech samples (40 analysis samples and 20 repetition samples) presented by the researcher and recorded their responses in a Google form. More specifically, in stage 1, the students rated the 60 speech samples (40 analysis samples and 20 repetition samples) according to their own criteria and then filled in the corresponding responses on the form. In stage 2, the students re-rated the same speech samples in stage 1, following their own criteria and then filled in the corresponding responses on the form. A total of 120 samples were analyzed by each student in the study. After every 20 minutes of sample presentation, a five-minute break was given to avoid fatigue. After confirming receipt of the forms containing the analyzed data, the students were released from the video call. The internet connection of the participants and the researcher remained stable throughout the completion of the proposed activities.

### Data analysis method

The results regarding the classification of hypernasality were initially presented using descriptive statistics, with the percentage (%) of correct answers according to the gold-standard evaluation for each evaluator, within each group (G1 or G2), considering both scales. The percentage of intra-evaluator agreement within each group was also presented, considering both scales.

To test the hypothesis that the classification of speech hypernasality using the 3-point scale would result in superior performance (higher percentage of correct answers in relation to the gold standard) in the degree of hypernasality, compared to the 4-point scale, a comparison of the mean percentage of correct answers was performed using mixed repeated measures ANOVA. The analysis considered the effects of group (order of presentation), factor (scale) and the interaction between group and factor. To test the hypothesis that the classification of speech hypernasality by the same evaluator (intra-evaluator agreement) using the 3-point scale would result in superior performance compared to the 4-point scale, a comparison of the mean percentage of agreement was performed using mixed repeated measures ANOVA. The analysis considered the effects of group (order of presentation), factor (scale) and the interaction between group and factor. The verification of the homogeneity of variances for the ANOVA was performed using the Levene test and post-hoc comparisons were conducted using the Bonferroni test. The Kappa coefficient (k) was also calculated to analyze the students' agreement in relation to the gold-standard assessment, as well as to evaluate the intra-evaluator agreement on both scales. The Kappa coefficient (k) values ​​were interpreted, according to Landis and Koch^(23)^, as below 0 indicating no agreement, from 0 to 0.19 indicating poor agreement, from 0.20 to 0.39 fair agreement; from 0.40 to 0.59 moderate agreement; from 0.60 to 0.79 substantial agreement; from 0.80 to 1.00 almost perfect agreement. To compare the students' agreement rates in relation to the gold-standard assessment, the differences between the scales (3 and 4 points) for the Kappa coefficient (k) were analyzed by 95% confidence intervals. The comparison of the intra-rater agreement rates between the two scales was performed through descriptive analysis. All analyses were conducted using SPSS software (version 24.0) for Windows, with a significance level set at 5% (p<0.05).

## RESULTS

### Comparing student analyses to the gold-standard assessment

The mean values ​​and standard deviations for the percentage of correct answers given by the students, using their own criteria, in relation to the gold- standard assessment, both for the total number of participants (20 students) and for each individual group (10 students), in each of the scales, are summarized in Table 1. There was a main effect for the factor (scale) (p=0.007), regardless of the group. The absence of a significant interaction between group and factor indicates that the order of the classifications (group) did not influence the results. A significant difference was observed in the mean of the total percentage of correct answers given by the students between the two scales, with higher values ​​for the 3-point scale.

**Table 1:** Comparison of mean and standard deviation for percentage of correct answers with the gold-standard assessment by group (G1=10 students; G2=10 students) and scale (factor)

**Variable**	**Group**	**Scale 4 pts**		**Scale 3 pts**		**Anova (p-value)**		
		**Average**	**DP**	**Average**	**DP**	**Group**	**Factor**	**Interaction**
% correct	G1	57,27	11.29	67.00	6.85	0.923	0.007*	0.560
	G2	58.55	10.72	65.10	7.32			
	Total	57.91	10.73	66.05ǂ	6.97			

Note: *indicates significant effect of the scale (factor) by the repeated measures ANOVA test regardless of the group for p-value ≤ 0.050; ǂindicates significant difference in relation to the 4-point scale (pts) by the Bonferroni Post-Hoc test for p-value ≤ 0.050

The agreement between the students' analyses and the gold-standard assessment, together with the respective Kappa coefficient (k) indices of the 20 grouped students, was established for each of the scales (Table 2). The results revealed that, for the 4-point scale, the total Kappa coefficient index of the students was 0.375, interpreted as fair agreement, being statistically significant (p<0.001). For the 3-point scale, this index was 0.491, considered as moderate agreement, also being statistically significant (p<0.001). When comparing the findings obtained between the 3-point and 4-point scales, a significant increase in the agreement index for the 3-point scale was observed (95% confidence interval analysis).

**Table 2:** Analysis of student agreement (n=20) and gold standard for a 4 and 3 point scale

**Group (students)**	**Category**	**Kappa**	**IC95%**		**p-value**
			**LI**	**LS**	
Score 4 pts	1	0.525	0.503	0.546	<0.001*
	2	0.273	0.252	0.295	<0.001*
	3	0.286	0.265	0.308	<0.001*
	4	0.460	0.439	0.481	<0.001*
	Total	0.375	0.363	0.388	<0.001*
Score 3 pts	1	0.525	0.504	0.547	<0.001*
	2	0.323	0.302	0.345	<0.001*
	3	0.622	0.600	0.643	<0.001*
	Total	0.491	0.476	0.507	<0.001*

Note: 4-pt scale (categories): 1=absent; 2=mild hypernasality; 3=moderate hypernasality; 4=severe hypernasality; 3-pt scale (categories): 1=absent; 2=slightly hypernasal; 3=very hypernasal

**Caption:** pts=points; LI=lower limit and LS=upper limit; Analysis of 95% confidence intervals; *significant kappa coefficient for p-valor ≤0.05

### Agreement of intra-rater analyses

The mean values ​​and standard deviations for the percentage of agreement in the analyses of duplicate samples for the total number of participants and for each individual group, in each of the scales, are summarized in Table 3. There was a main effect for the factor (scale), regardless of the group (p=0.006). The absence of a significant interaction between the group (order of presentation) and the factor (scale) indicates that the order of presentations (groups) did not influence the results. A significant difference was observed in the mean total agreement in the analyses of the students between the two scales, with higher values ​​for the 3-point scale.

**Table 3:** Comparison of mean and standard deviation for percentage of intra-rater agreement by group and scale (factor)

**Variable**	**Group**	**Scale 4 pts**		**Scale 3 pts**		**Anova (p-value)**		
		**Average**	**DP**	**Average**	**DP**	**Group**	**Factor**	**Interaction**
% agreement	G1	55.00	13.33	71.50	15.47	0.892	0.006*	0.766
	G2	52.50	21.63	72.50	19.61			
	Total	53.75	17.54	**72.00**ǂ	17.20			

Note: *indicates significant effect of the scale (factor) by the repeated measures ANOVA test regardless of the group for p-value ≤ 0.050;

ǂ indica diferença significativa em relação a escala de 4 pontos (pts) pelo teste Post-Hoc de Bonferroni para p-valor ≤0,050

The absolute and relative distribution of intra-rater agreement, based on the Kappa coefficient (k) indices of each rater and their respective interpretations, obtained for each scale (3 and 4 points), are presented in Table 4. In the 4-point scale, there was no agreement in the responses of one evaluator (5%). Agreement was moderate for 15% of the evaluators, substantial or poor for 25% and slight for 30%. Perfect/near-perfect agreement was not achieved using this scale. On the 3-point scale, perfect/near-perfect agreement was observed for 20% of the evaluators, slight or substantial for 20% and moderate for 35%. Poor agreement was obtained for only one evaluator (5%).

**Table 4:** Absolute and relative distribution of intra-rater agreement. according to Kappa interpretation. for the 4-point and 3-point scales

**Kappa Interpretation**	**4 points**	**%**	**3 points**	**%**
No agreement	1	5	-	-
Poor	5	25	1	5
Slight	6	30	4	20
Moderate	3	15	7	35
Substantial	5	25	4	20
Perfect/near perfect	-	-	4	20
Total	20	100	20	100

## DISCUSSION

In the study, when comparing the results of the hypernasality classification using the two scales, the mean number of correct responses from students was significantly higher for the 3-point scale than for the 4-point scale, in relation to the gold-standard evaluation. This suggests that the reduced scale favored the classification of hypernasality by students. These findings corroborate the arguments of previous research scholars^(16)^, who proposed the use of a three-point scale for the classification of hypernasality by listeners without experience in this task, since the use of the scale with higher degrees can generate an adverse interaction with the scale itself when used by these listeners. Previous studies also refer to the use of a three-point scale in speech classifications, including by speech therapists experienced in speech management in CLP/VDF^(17,18)^.

In the present study, inferential analyses revealed that there was no significant interaction between group (order of presentation) and factor (scale grade) for the mean percentage of correct classifications. This suggests that the order of classifications performed using the scales did not influence the results. In other words, regardless of the order in which the classifications were performed (either first with the three-point scale or first with the four-point scale), the results were more favorable for the classifications performed using the three-point scale (reduced) by untrained listeners. All students participating in the study were inexperienced listeners, that is, students in the initial years of the Speech-Language Pathology course, with no previous involvement in treating patients with CLP and/or VDF. Regarding the level of knowledge about CLP and associated speech disorders, information derived from a questionnaire showed that 83% of the students had no knowledge about CLP, VDF or hypernasality. The remainder (17% of students) stated that they had some information about CLP or had briefly heard speech alterations related to CLP/VDF, but were unable to distinguish them. Considering that none of the listeners had experience in assessing hypernasality, it was assumed that they would benefit from using a scale with less variability in degrees to classify speech hypernasality, a fact confirmed by the present investigation.

A previous study^(22)^ found a percentage of 62.5% of correct responses in relation to the gold-standard in the classification of hypernasality in speech samples containing only oral sounds (high pressure), performed by inexperienced speech therapists, using the 4-point scale^(22)^. When comparing this percentage with the findings of the present study, a lower value was observed for the 4-point scale and a higher value for the 3-point scale. This suggests that, for students without any clinical experience, the reduced scale was favorable, while the 4-point scale made the analyses more difficult.

In a previous study^(24)^, in which hypernasality was classified by students in the initial years of a Speech Therapy course, a percentage of 66% of correct answers was obtained in relation to the gold-standard evaluation for the analysis of low-pressure oral samples, using the 4-point scale. In the present study, a lower percentage was obtained for high-pressure samples in the analyses performed by the students, also using the 4-point scale. The inclusion of samples consisting of a set of 12 high-pressure sentences^(9)^ may have made it difficult for students to classify hypernasality using the 4-point scale, due to other coexisting speech alterations, such as nasal air emission and/or compensatory articulations. On the other hand, results similar to those of the previous study^(25)^ were obtained in the present study, using the 3-point scale, suggesting that this scale favored the analyses when coexisting speech alterations were not controlled. Some studies propose the inclusion of reference samples in perceptual analyses performed to assess speech disorders related to CLP/VDF by both inexperienced^(20,22)^ and experienced^(6)^ speech therapists. The references aim to favor and increase the consistency of responses, since instability in the internal standards of the evaluators can be minimized by perceptual references, promoting greater agreement between listeners(6). In a previous study^(16)^ involving inexperienced listeners, researchers offered two reference samples (one with the absence and the other with the presence of severe hypernasality). These samples were presented to the listeners before the judgment task and repeated every five samples, before the listeners (children) analyzed the samples using the three-point scale.

Unlike the previous study mentioned^(16)^, in the present study, no reference or training samples were made available that could interfere with the analyses performed by the students using the two proposed scales (four and three points). Before starting the analyses, the researcher only gave a brief PowerPoint presentation to the students, addressing definitions of CLP< VDF and hypernasality, with the aim of familiarizing them with the topics, the speech aspect to be evaluated and the scales. The procedures used in the present study focused exclusively on verifying the effect of the scales on the analyses of inexperienced listeners, without interference from perceptual references. This study also verified the Kappa agreement indices (and their interpretation) of the students' analyses in comparison with the gold-standard evaluation, using the four and three-point scales. The results revealed fair agreement for the four-point scale and moderate agreement for the three-point scale, indicating greater reliability in the analyses performed with the three-point scale. Furthermore, the significant increase in the agreement rate for the 3-point scale (based on the 95% confidence interval) compared to the 4-point scale, as observed in this study, suggests that the reduced scale favored the reliability of the students' analyses in relation to the gold-standard assessment. On the other hand, the findings of the study show that the use of the 4-point scale by inexperienced listeners can hinder perceptual analyses of the degrees of hypernasality, which was also noted in a previous investigation^(22)^.

The moderate agreement found in the study (three-point scale) can be explained by the characterization of the listeners (inexperienced students), the methodological procedures employed (no inclusion of reference samples) and the speech samples included (no control for the coexistence of other speech alterations). According to scholars, hypernasality, when associated with compensatory articulations (use of atypical articulation point), can be perceptually judged as more nasalized^(10)^. Although the speech samples in this study were controlled for dysphonia due to its possible impact on hypernasality classification^(26)^, other speech symptoms such as nasal air emission/turbulence were also not controlled and, therefore, may have impacted the agreement of the evaluators' analyses, in relation to the gold-standard evaluation.

One of the objectives of this study was to investigate the percentage of agreement of the intra-evaluator analyses by group and scale. The results revealed a significantly higher mean percentage of agreement of the duplicate analyses for the 3-point scale (72%) than for the 4-point scale (53.75%). The analyses performed also indicated that there was no significant interaction between group (order of presentation) and factor (scale grade) for the percentage of intra-evaluator classifications, suggesting again that the order of the classifications performed using the scales did not influence the results of the intra-evaluator agreement. It can be observed, therefore, that the three-point scale favored the agreement of the intra-evaluator analyses.

Intra-rater Kappa indices (and their respective interpretations) were also obtained using the two scales. The absolute and relative distribution of intra-rater agreement, based on the Kappa interpretation, revealed discrepant results for the four-point and three-point scales. On the four-point scale, there was no agreement in the responses of one rater (5%). There was moderate agreement for 15% of the raters, poor agreement for 25% of the raters, and substantial agreement for another 25%, with a higher percentage of slight agreement (30% of the raters).

In contrast, for the three-point scale, no disagreements were recorded. There was perfect/near-perfect agreement for 20% of the raters. In addition, 20% of the raters showed slight or substantial agreement. There was a higher percentage of moderate agreement (35%), while poor interpretation occurred for only one rater (5%). Taken together, these findings suggest a more favorable intra-rater agreement for the three-point scale, indicating greater reliability in the intra-rater analyses for this scale.

Discussions about procedures that can favor the auditory-perceptual analysis of speech hypernasality are frequent among scholars^(5,24)^. Some express reservations regarding the validity of categorical scales in the analysis of speech characteristics for clinical and research purposes^(8,12,13,27)^, arguing that listeners do not perceive exactly equal intervals (or categories) during their analyses^(26)^, tending to subdivide the lower end of the scale into smaller intervals^(13)^. However, scales with equal or ordinal intervals have been the most used resource by clinicians and researchers^(9,20,28)^, especially in clinical routine, since these scales seem to be intuitively easy to apply, allowing the comparison of findings between scales and evaluators^(14)^.

The results of this study indicate that, when using categorical scales in perceptual analyses, the 3-point scale may favor the classification of hypernasality by inexperienced listeners, in agreement with previous findings^(16)^. It is recommended the reduced scale to be initially adopted in clinical experiences with students in training. The use of scales with fewer degrees or categories may benefit the resolution task in perceptual analyses.

The present study contributes significantly to a better understanding of the influence of the scales' scoring level on perceptual analyses performed by inexperienced listeners, i.e., students in training without knowledge or clinical experience related to speech alterations in CLP/VDF. As highlighted in a previous study^(16)^, reduced categorical scales can improve the reliability of hypernasality classification by untrained listeners. In this sense, it is necessary to continue conducting studies that employ reduced scales for these listeners.

This argument is based on the findings of previous studies that indicated little or no effect of brief auditory-perceptual training aimed at classifying speech hypernasality using the 4-point scale by untrained Speech-language Pathologists^(22)^ or otorhinolaryngology residents^(29)^. It is suggested that, in addition to structured and longer training, the implementation of the reduced scale as part of such training may improve the perceptual classifications of these listeners.

Although the experimental task of the study was supervised by the researcher, the students used their own headphones. The researcher monitored all students to ensure that they used the same headphones on both days of analysis, taking place in a noise-free environment. However, in future studies, it is recommended controlled headphones to be used and the assessments to be carried out in the same location.

It is worth mentioning that audio recordings of speech with quality equipment for analysis by multiple evaluators and standardization of speech stimuli were controlled in the study, since they are considered important strategies to achieve reliability of perceptual analyses (20). However, there was no control for coexisting alterations in the speech samples, which may have impacted the classification of hypernasality in both scales. For future studies, it is suggested to compare the analysis between scales of different degrees, considering the control of other speech symptoms related to CLP/VDF.

## CONCLUSION

The three-point scale provided a better classification of the degree of speech hypernasality by students in a Speech Therapy course. The average percentage of correct responses compared to the gold-standard assessment was significantly higher for this scale when compared to the results obtained with the four-point scale. The analysis of intra-rater agreement revealed significant differences between the two scales, with a higher percentage of intra-rater concordant responses for the three-point scale. The order in which the scales were presented did not influence the classifications made. The analyses based on the Kappa statistics demonstrated greater intra-rater agreement in the classifications made with the three-point scale.
