# Supplementary material for: Classification of speech nasality of individuals with cleft lip and palate with distinct ordinal scales
Source: Codas. 2025 Jan 27;37(1):e20240044. doi: 10.1590/2317-1782/e20240044en (PMC11781359; doi:10.1590/2317-1782/e20240044en)
Supplement: Translation

# Translation: Classificação da nasalidade de fala de indivíduos com fissura labiopalatina com escalas ordinais distintas

**DOI:** 10.1590/2317-1782/e20240044pt

**Authors:** Gisele Fonseca do Carmo, Jeniffer de Cássia Rillo Dutka, Flora Taube Manicardi, Beatriz Campanine Geremias, Maria Inês Pegoraro-Krook, Viviane Cristina de Castro Marino

**Affiliations:** 1 Programa de Pós-Graduação em Fonoaudiologia, Universidade Estadual Paulista “Júlio de Mesquita Filho” – UNESP - Marília (SP), Brasil.; 2 Pós-Graduação em Ciência da Reabilitação, Hospital de Reabilitação de Anomalias Craniofaciais, Universidade de São Paulo – USP - Bauru (SP), Brasil

**Keywords:** Fissura Palatina, Insuficiência Velofaríngea, Distúrbios da Fala, Percepção da Fala, Fala

## Abstract

**Objetivo:**

Investigar se há diferença na classificação da hipernasalidade de fala de ouvintes não experientes usando escalas ordinais distintas; verificar a concordância dos ouvintes nas análises ao empregar estas escalas e verificar se a ordem de apresentação das escalas influencia os resultados.

**Método:**

Vinte acadêmicos em Fonoaudiologia classificaram os graus de hipernasalidade de 40 amostras (orais) de pacientes com fissura labiopalatina. Dez realizaram as classificações utilizando escala de 4 pontos (ausente, leve, moderada e grave) e, após duas semanas, utilizando escala de 3 pontos (ausente, pouco hipernasal e muito hipernasal). Outros dez alunos realizaram as mesmas classificações, porém em ordem inversa. As classificações foram feitas de forma remota e documentadas em um formulário.

**Resultados:**

A porcentagem média de respostas corretas dos alunos, em relação ao padrão-ouro, foi significativamente maior para a escala de 3 pontos. Não houve interação significativa entre ordem de apresentação e escala para o percentual de acerto das classificações. A concordância dos alunos em relação à avaliação padrão-ouro foi regular (escala de 3 pontos) e moderada (escala de 4 pontos). A porcentagem média de concordância das análises intra-avaliador foi significativamente maior para a escala de 3 pontos. Não houve interação significativa entre ordem de apresentação e escala para o percentual das classificações intra-avaliadores. O índice de coeficiente Kappa mostrou concordância intra-avaliador mais favorável para a escala reduzida.

**Conclusão:**

A escala reduzida favoreceu a classificação da hipernasalidade de fala pelos ouvintes e pode ser considerada uma importante estratégia para favorecer as avaliações iniciais de acadêmicos em Fonoaudiologia durante sua formação.

## INTRODUÇÃO

A hipernasalidade é um importante sintoma de fala na presença de disfunção velofaríngea (DVF) após a correção cirúrgica do palato^(1,2)^. A avaliação perceptivo-auditiva é essencial para identificar este sintoma de fala^(3)^. Por meio dessa avaliação, clínicos conseguem identificar e mensurar a gravidade da hipernasalidade^(4)^, o que facilita a tomada de decisões clínicas^(3)^, além de permitir o acompanhamento dos resultados alcançados^(5)^. No entanto, essa avaliação é baseada nas impressões auditivas do ouvinte^(5)^ e suscetível a erros e vieses, mesmo quando realizada por profissionais experientes^(1,3,6)^.

Avaliar de modo confiável as características de fala relacionadas à DVF é um processo desafiador, uma vez que variações nos resultados das avaliações perceptivas da hipernasalidade podem ser justificadas pelo grau de experiência clínica e pelos critérios adotados por cada avaliador em suas análises^(5)^. Outras variáveis que podem influenciar na avaliação perceptivo-auditiva, afetando sua confiabilidade, incluem o tipo, a extensão e o contexto fonético do estímulo de fala^(7-9)^ e, também, a presença de alterações coexistentes^(10).^

Várias estratégias são recomendadas para reduzir os vieses da subjetividade presentes nesse método de avaliação, destacando-se o estabelecimento de critérios de escores (por meio do uso de escalas) para a classificação da hipernasalidade^(5,8)^. O uso de escalas adequadas emerge como uma estratégia crucial para aprimorar a confiabilidade na classificação da hipernasalidade^(1,3,8,11)^. Diferentes tipos de escalas são descritos na literatura, abrangendo desde escalas de intervalos iguais ou ordinais, estimativa de magnitude direta, escala visual analógica^(5)^ até a escala de Borg^(8,12)^.

O debate em torno dos tipos de escalas e métodos que podem ser empregados para aprimorar a avaliação perceptivo-auditiva continua entre os pesquisadores ^(5).^ As escalas de proporção, especialmente a escala Borg, oferecem a perspectiva de ampliar a confiabilidade nas classificações da hipernasalidade por diferentes avaliadores^(8,12)^. No entanto, essas escalas apresentam desvantagens para aplicações clínicas. A escala de estimativa direta, por exemplo, é considerada impraticável para o uso rotineiro na clínica por exigir um treinamento mais extenso dos avaliadores^(13)^. Em contrapartida, as escalas com intervalos iguais são as mais frequentemente empregadas na avaliação perceptivo-auditiva^(6,9)^ e consideradas as mais adequadas para serem utilizadas em contexto clínico por possibilitarem a obtenção de classificações pelos avaliadores de forma relativamente fácil^(14)^, permitindo a comparação dos resultados entre escalas e avaliadores.

Na escala intervalar, o avaliador atribui um índice (ou categoria) para o aspecto de fala avaliado, indicando o seu nível de gravidade. O menor valor (categoria) refere-se à ausência da alteração enquanto o maior valor (categoria) indica o grau máximo da alteração^(15)^. A literatura indica variabilidade quanto à pontuação a ser utilizada pelas escalas categóricas para classificar a hipernasalidade, podendo variar em três pontos^(16-18)^, quatro pontos^(19,20)^, cinco pontos^(4,12)^ ou até mesmo em mais pontos^(21)^. A escala de quatro pontos é frequentemente empregada, seguindo as recomendações dos parâmetros universais para documentação da fala na fissura labiopalatina (FLP)^(11)^.

Numa tentativa de minimizar as dificuldades associadas à tarefa de classificar a hipernasalidade de fala por ouvintes sem experiência, como crianças, alguns estudiosos^(16)^ propuseram o uso de escala de três pontos (normal, um pouco hipernasal e muito hipernasal) ao invés de escala com maior número de opções. Argumentaram que o uso de escalas com mais opções pode resultar em avaliações menos confiáveis entre ouvintes não experientes devido a possível interação adversa com a própria escala. Em estudo anterior^(16)^ os resultados mostraram que as classificações da hipernasalidade feitas pelas crianças foram compatíveis com aquelas realizadas pela profissional experiente. Segundo os autores, a justificativa para esses achados está relacionada à utilização da escala reduzida, a qual, por oferecer menor quantidade de opções, facilitou as respostas das crianças. Dois outros estudos também empregaram escalas ordinais de três pontos para classificar a hipernasalidade, porém, as classificações foram realizadas por fonoaudiólogos experientes^(17,18)^. Observa-se, portanto, que apenas três estudos utilizaram escalas categóricas reduzidas (três pontos) para a classificação da hipernasalidade de fala, sendo que somente um deles envolveu ouvintes sem experiência nas análises.

Dada a natureza subjetiva da avaliação perceptivo-auditiva e considerando que escalas com maior número de pontos (por exemplo, quatro pontos) podem complicar a classificação da hipernasalidade por ouvintes não experientes, surge a questão de se os acadêmicos em Fonoaudiologia poderiam se beneficiar ao utilizar uma escala reduzida, conforme proposto em estudo prévio^(16)^. Assim, os objetivos do estudo foram (1) investigar se há diferença na classificação da hipernasalidade de fala de indivíduos com FLP por ouvintes não experientes (acadêmicos de Fonoaudiologia) usando escalas ordinais distintas (3 e 4 pontos), (2) verificar a concordância dos ouvintes nas análises ao empregar estas duas escalas e (3) verificar se a ordem de apresentação das escalas influencia os resultados.

## MÉTODO

O estudo recebeu aprovação do Comitê de Ética em Pesquisa com Seres Humanos do local onde foi conduzido (parecer nº 5.679.783). Todos os participantes que concordaram em participar do estudo assinaram o Termo de Consentimento Livre e Esclarecido. Foram utilizadas gravações de fala pré-existentes de indivíduos com histórico de FLP, de ambos os sexos. Posteriormente, essas gravações foram avaliadas por 20 participantes, empregando escalas de intervalos distintos.

### Amostras de fala

As gravações de fala foram selecionadas a partir de um conjunto pré-existente em uma base de dados e foram originalmente obtidas diretamente do computador, equipado com placa de som *Sound Blaster Audigy 2* e programa Sony® Sound Forge, versão 8.0, com taxa de amostragem de 44100 Hz, em monocanal, 16 Bits. O sinal áudio foi capturado utilizando microfone de cabeça (modelo AKG C420®), em sala acusticamente tratada. Todas as gravações do estudo tinham boa qualidade de áudio, com um intervalo padronizado de um segundo entre cada uma das frases. O estímulo de fala compreendeu um conjunto de 12 frases constituídas predominantemente por fonemas de alta pressão intraoral, em que cada frase era composta por um único som-alvo em recorrência, seguindo a metodologia de estudos prévios^(9,22).^

No estudo, foram incluídas 40 amostras de fala (em áudio) de indivíduos com FLP. As amostras de fala foram selecionadas de uma base de dados de gravações previamente agrupadas de acordo com o grau de nasalidade. A primeira autora do estudo ouviu as gravações e as primeiras 10 amostras de fala com boa qualidade de gravação que foram consideradas (pela autora) como representativas de cada um dos quatro graus de nasalidade (A = hipernasalidade ausente, HL = hipernasalidade leve, HM = hipernasalidade moderada e HG = hipernasalidade grave) foram selecionadas para o estudo. As amostras de fala selecionadas não apresentavam disfonia. Outros sintomas de fala, como emissão de ar nasal/turbulência, articulação compensatória, não foram controlados no presente estudo.

### Avaliação perceptiva padrão-ouro

As gravações selecionadas para o estudo foram analisadas por fonoaudiólogas experientes em dois momentos. O grau de hipernasalidade na escala de 4 pontos foi pré-estabelecido, conforme descrito em estudo anterior^(22)^ e possibilitou a criação de um banco de dados no qual a primeira autora selecionou as 40 gravações incluídas no estudo, sendo 10 (25%) constituídas de amostras de fala representativas de HA, 10 (25%) constituídas de amostras de fala representativas de HL, 10 (25%) constituídas de amostras de fala representativas de HM e 10 (25%) constituídas de amostras de fala representativas de HG. A primeira autora, ao selecionar as 40 amostras de fala, concordou com a avaliação padrão-ouro reportada em estudo prévio^(22)^.

A escala de 3 pontos, no entanto, ainda não havia sido aplicada nas 40 gravações selecionadas. O procedimento de análises das gravações, aplicando-se a escala de 3 pontos (A = hipernasalidade Ausente, PH = pouca hipernasalidade e MH, muita hipernasalidade) foi realizado para o presente estudo por fonoaudiólogas experientes, sendo que as mesmas 40 gravações previamente selecionadas foram reagrupadas, sendo 23 (30%) constituídas por amostras representativas de A, 9 (22,5%) por amostras representativas de PH e 19 (47,5%) por amostras representativas por MH. Ao reagrupar as gravações, houve concordância nas análises de pelo menos duas de três fonoaudiólogas experientes, sem discordâncias entre elas. Todas as fonoaudiólogas que realizaram análises de amostras de fala incluídas no estudo participam rotineiramente de avaliações de fala de pacientes com FLP e atuam em um mesmo centro de gerenciamento de anomalias craniofaciais. As análises das amostras de fala realizadas pelas especialistas, usando cada uma das escalas, foram adotadas como critério padrão-ouro para comparações com as avaliações dos participantes.

### Participantes

Vinte alunos, com idades entre 20 e 40 anos (média de 21 anos e 3 meses), provenientes de um Curso de Graduação em Fonoaudiologia e falantes nativos do Português Brasileiro, participaram deste estudo. Todos os alunos estavam matriculados no segundo ano de graduação e ainda não tinham cursado a disciplina teórica na área da FLP. Ademais, todos os participantes afirmaram possuir audição normal e nenhuma experiência prévia na avaliação de fala de indivíduos com FLP.

### Procedimentos

Os 20 participantes do estudo foram distribuídos em dois grupos: Grupo 1 (G1) e Grupo 2 (G2), cada um composto por 10 alunos, selecionados de maneira aleatória para conduzir as análises das amostras de fala utilizando as duas escalas propostas. Todos os participantes dos Grupos G1 e G2 realizaram a classificação de 40 amostras de fala, incluindo também, 20 amostras duplicadas para uma subsequente análise da concordância intra-avaliador, totalizando 60 amostras de fala analisadas com o uso de cada uma das escalas. A apresentação de todas as amostras ocorreu de forma aleatória.

Os alunos dos grupos G1 e G2 conduziram as análises das amostras de maneira individual por meio de uma reunião online, utilizando seus próprios fones de ouvido. As análises foram realizadas em duas etapas distintas. Na Etapa 1, os participantes do G1 realizaram a classificação das 40 amostras de fala (além de 20 amostras de repetição) utilizando a escala de 4 pontos (A, HL, HM, HG) enquanto participantes do G2 realizaram a classificação das mesmas 40 amostras de fala (juntamente com 20 de repetição), porém utilizando a escala de 3 pontos (A, PH, MH). Na etapa 2, participantes do G1 reclassificaram as mesmas 40 amostras de fala (mais 20 de repetição) utilizando a escala de 3 pontos (A, PH, MH). Por outro lado, participantes do G2 realizaram essa tarefa, porém, utilizando a escala de 4 pontos (A, HL, HM, HG). Entre as duas etapas do estudo houve um intervalo de 2 semanas.

Em cada etapa do estudo, as amostras de fala foram apresentadas pela primeira autora (G. F. C.), por meio da plataforma Google Meet, para um ou mais alunos que constituíram cada grupo. No total, cada aluno participou de dois encontros, com uma média de duração de 70 minutos por encontro. A duração de cada sessão incluiu o tempo dedicado para que o aluno ou grupo de alunos ingressasse na videoconferência; a apresentação em PowerPoint seguida da oferta de instruções técnicas pela pesquisadora; o período destinado a esclarecer possíveis dúvidas e, também, o tempo de pausas pré-estabelecido durante a coleta.

Foi disponibilizada uma apresentação em *PowerPoint* para os alunos de cada grupo antes da realização das atividades. Esta apresentação incluía definições concisas sobre FLP, DVF e hipernasalidade, com o objetivo de familiarizar os alunos com o tema. Não foram incluídas amostras de referências nem informações que pudessem influenciar suas análises. Os alunos foram informados de que cada amostra de fala seria apresentada uma vez, com a opção de repetição, caso necessário. Eles também receberam instruções para utilizar o mesmo fone de ouvido e estarem no mesmo ambiente silencioso durante ambas as etapas do estudo.

Em cada etapa do estudo, os alunos ouviram as 60 amostras de fala (40 amostras de análise e 20 amostras de repetição) apresentados pela pesquisadora e registraram suas respostas em um formulário Google. Mais especificamente, na etapa 1 os alunos classificaram as 60 amostras de fala (40 amostras de análise e 20 amostras de repetição), seguindo seus próprios critérios e, em seguida, preencheram as respostas correspondentes no formulário. Na etapa 2, os alunos reclassificaram as mesmas amostras de fala na etapa 1, seguindo seus próprios critérios e, em seguida, preencheram as respostas correspondentes no formulário. Um total de 120 amostras foram analisadas por cada aluno no estudo. A cada 20 minutos de apresentação das amostras, foi concedido um intervalo de cinco minutos para evitar fadiga. Após a confirmação de recebimento dos formulários contendo os dados analisados, os alunos foram liberados da chamada de vídeo. A conexão de internet dos participantes e da pesquisadora permaneceu estável durante toda a realização das atividades propostas.

### Forma de análise dos dados

Os resultados referentes à classificação da hipernasalidade foram inicialmente apresentados por estatística descritiva, com a porcentagem (%) de acertos de acordo com a avaliação padrão-ouro para cada avaliador, dentro de cada grupo (G1 ou G2), considerando ambas as escalas. Também foram apresentadas a porcentagem de concordância intra-avaliador dentro de cada grupo, considerando ambas as escalas.

Para testar a hipótese de que a classificação da hipernasalidade de fala, utilizando a escala de 3 pontos, resultaria em desempenho superior (maior porcentagem de acertos em relação ao padrão-ouro) no grau de hipernasalidade, em comparação com a escala de 4 pontos, realizou-se uma comparação do percentual médio de acertos através da Anova de medidas repetidas mista. A análise considerou os efeitos do grupo (ordem de apresentação), fator (escala) e a interação entre grupo e fator. Para testar a hipótese de que a classificação da hipernasalidade de fala pelo mesmo avaliador (concordância intra-avaliador) usando a escala de 3 pontos, resultaria em desempenho superior em comparação com a escala de 4 pontos, realizou-se uma comparação do percentual médio de concordância por meio da Anova de medidas repetidas mista. A análise considerou os efeitos do grupo (ordem de apresentação), fator (escala) e a interação entre grupo e fator. A verificação da homogeneidade das variâncias para a Anova foi realizada por meio do teste de Levene e as comparações pós-hoc foram conduzidas utilizando o teste de Bonferroni.

O coeficiente Kappa (k) também foi calculado para analisar a concordância dos alunos em relação à avaliação padrão-ouro, bem como para avaliar a concordância intra-avaliador em ambas as escalas. Os valores do coeficiente Kappa (k) foram interpretados, conforme Landis e Koch^(23)^ abaixo de 0 indicando ausência de concordância; de 0 a 0,19 concordância pobre; de 0,20 a 0,39 concordância regular; de 0,40 a 0,59 concordância moderada; de 0,60 a 0,79 concordância substancial; de 0,80 a 1,00 concordância quase perfeita. Para comparar os índices de concordância dos alunos em relação à avaliação padrão-ouro, as diferenças entre as escalas (3 e 4 pontos) para o coeficiente Kappa (k) foram analisadas pelos intervalos de confiança de 95%. A comparação dos índices de concordância intra-avaliadores entre as duas escalas foi realizada por meio de análise descritiva. Todas as análises foram conduzidas utilizando o software SPSS (versão 24.0) para Windows, com um nível de significância estabelecido em 5% (p<0,05).

## RESULTADOS

### Comparação das análises dos alunos com a avaliação padrão-ouro

Os valores médios e os desvios-padrão referentes à porcentagem de acertos nas classificações dos alunos, por meio dos critérios próprios, em relação à avaliação padrão-ouro, tanto para o total de participantes (20 alunos) quanto para cada grupo individual (10 alunos), em cada uma das escalas, são sumarizados na Tabela 1. Houve efeito principal para o fator (escala) (p=0,007), independente do grupo. A ausência de uma interação significativa entre grupo e fator indica que a ordem das classificações (grupo) não influenciou os resultados. Foi observada uma diferença significativa na média da porcentagem total de acertos nas classificações dos alunos entre as duas escalas, com valores superiores para a escala de 3 pontos.

**Tabela 1:** Comparação de média e desvio-padrão para porcentagem de acerto com a avaliação padrão-ouro por grupo (G1=10 alunos; G2=10 alunos) e escala (fator)

**Variável**	**Grupo**	**Escala 4 pts**		**Escala 3 pts**		**Anova (p-valor)**		
		**Média**	**DP**	**Média**	**DP**	**Grupo**	**Fator**	**Interação**
% acerto	G1	57,27	11,29	67,00	6,85	0,923	0,007*	0,560
	G2	58,55	10,72	65,10	7,32			
	Total	57,91	10,73	66,05ǂ	6,97			

Nota: *indica efeito significativo da escala (fator) pelo teste de Anova de medidas repetidas independente do grupo para p-valor ≤ 0,050 ǂindica diferença significativa em relação a escala de 4 pontos (pts) pelo teste Post-Hoc de Bonferroni para p-valor ≤0,050

A concordância das análises dos alunos em relação à avaliação padrão-ouro, juntamente com os respectivos índices de coeficiente Kappa (k) dos 20 alunos agrupados, foi estabelecida para cada uma das escalas (Tabela 2). Os resultados revelaram que, para a escala de 4 pontos, o índice total de coeficiente Kappa dos alunos foi de 0,375, interpretado como concordância regular, sendo estatisticamente significativo (p<0,001). Já para a escala de 3 pontos, este índice foi de 0,491, considerado como concordância moderada, também sendo estatisticamente significativo (p<0,001). Ao comparar os achados obtidos entre as escalas de 3 e 4 pontos, observou-se um aumento significativo no índice de concordância para a escala de 3 pontos (análise do intervalo de confiança de 95%).

**Tabela 2:** Análise da concordância dos alunos (n=20) e padrão-ouro para escala de 4 e 3 pontos

**Grupo (alunos)**	**Categoria**	**Kappa**	**IC95%**		**p-valor**
			**LI**	**LS**	
Escore 4 pts	1	0,525	0,503	0,546	<0,001*
	2	0,273	0,252	0,295	<0,001*
	3	0,286	0,265	0,308	<0,001*
	4	0,460	0,439	0,481	<0,001*
	Total	0,375	0,363	0,388	<0,001*
Escore 3 pts	1	0,525	0,504	0,547	<0,001*
	2	0,323	0,302	0,345	<0,001*
	3	0,622	0,600	0,643	<0,001*
	Total	0,491	0,476	0,507	<0,001*

Nota: escala 4 pts (categorias): 1= ausente; 2= hipernasalidade leve; 3= hipernasalidade moderada; 4=hipernasalidade grave; escala 3 pts (categorias): 1= ausente; 2=pouco hipernasal; 3=muito hipernasal

**Legenda:** pts=pontos; LI=limite inferior e LS=limite superior; Análise dos intervalos de confiança de 95%; *coeficiente kappa significativo para p-valor ≤0,05

### Concordância das análises intra-avaliadores

Os valores médios e os desvios-padrão referentes à porcentagem de concordância nas análises das amostras duplicadas para o total de participantes e para cada grupo individual, em cada uma das escalas, estão sumarizados na Tabela 3. Houve um efeito principal para o fator (escala), independente do grupo (p=0,006). A ausência de uma interação significativa entre o grupo (ordem de apresentação) e o fator (escala) indica que a ordem das apresentações (grupos) não influenciou os resultados. Observou-se uma diferença significativa na média de concordância total nas análises dos alunos entre as duas escalas, com valores superiores para a escala de 3 pontos.

**Tabela 3:** Comparação de média e desvio-padrão para porcentagem de concordância intra-avaliador por grupo e escala (fator)

**Variável**	**Grupo**	**Escala 4 pts**		**Escala 3 pts**		**Anova (p-valor)**		
		**Média**	**DP**	**Média**	**DP**	**Grupo**	**Fator**	**Interação**
% concordância	G1	55,00	13,33	71,50	15,47	0,892	0,006*	0,766
	G2	52,50	21,63	72,50	19,61			
	Total	53,75	17,54	**72,00**ǂ	17,20			

Nota: *indica efeito significativo da escala (fator) pelo teste de Anova de medidas repetidas independente do grupo para p-valor ≤ 0,050;

ǂ indica diferença significativa em relação a escala de 4 pontos (pts) pelo teste Post-Hoc de Bonferroni para p-valor ≤0,050

A distribuição absoluta e relativa da concordância intra-avaliador, com base nos índices de coeficiente Kappa (k) de cada avaliador e suas respectivas interpretações, obtidas para cada escala (3 e 4 pontos), estão apresentadas na Tabela 4. Na escala de 4 pontos, não houve concordância nas respostas de um avaliador (5%). A concordância foi moderada para 15% dos avaliadores, substancial ou pobre para 25% e leve para 30%. Não foi alcançada concordância perfeita/quase perfeita usando esta escala. Já na escala de 3 pontos, observou-se concordância perfeita/quase perfeita para 20% dos avaliadores, leve ou substancial para 20% e moderada para 35%. A concordância pobre foi obtida apenas para um avaliador (5%).

**Tabela 4:** Distribuição absoluta e relativa de concordância intra-avaliadores, de acordo com a interpretação Kappa, para as escalas de 4 pts e 3 pts

**Interpretação Kappa**	**4 pontos**	**%**	**3 pontos**	**%**
Sem concordância	1	5	-	-
Pobre	5	25	1	5
Leve	6	30	4	20
Moderada	3	15	7	35
Substancial	5	25	4	20
Perfeita/quase perfeita	-	-	4	20
Total	20	100	20	100

## DISCUSSÃO

No estudo, ao comparar os resultados da classificação da hipernasalidade utilizando as duas escalas, observou-se uma média de respostas corretas dos alunos, em relação à avaliação padrão-ouro, significativamente maior para a escala de 3 pontos do que para a escala de 4 pontos. Isto sugere que a escala reduzida favoreceu a classificação da hipernasalidade pelos alunos. Esses achados corroboram os argumentos de estudiosos de investigação prévia ^(16)^, que propuseram o uso de escala de três pontos para a classificação da hipernasalidade por ouvintes sem experiência nessa tarefa, uma vez que a utilização da escala de maiores graus pode gerar uma interação adversa com a própria escala quando utilizada por estes ouvintes. Estudos prévios também fazem referência ao uso de uma escala de três pontos em classificações da fala, inclusive por fonoaudiólogos experientes no gerenciamento da fala na FLP/DVF^(17,18)^.

No presente estudo, as análises inferenciais revelaram que não houve interação significativa entre o grupo (ordem de apresentação) e o fator (grau da escala) para o percentual médio de acerto das classificações. Isso sugere que a ordem das classificações realizadas utilizando as escalas não influenciou os resultados. Em outras palavras, independentemente da ordem em que as classificações foram realizadas (seja primeiro com a escala de três pontos ou primeiro com a escala de quatro pontos), os resultados foram mais favoráveis para as classificações feitas utilizando a escala de três pontos (reduzida) pelos ouvintes não treinados.

Todos os alunos participantes do estudo eram ouvintes não experientes, ou seja, estudantes das séries iniciais do curso de Fonoaudiologia, sem envolvimento prévio em atendimentos de pacientes com FLP e/ou DVF. Quanto ao nível de conhecimento sobre FLP e alterações de fala associadas, informações derivadas de um questionário mostraram que 83% dos alunos não tinham nenhum conhecimento sobre FLP, DVF ou hipernasalidade. O restante (17% alunos) afirmou ter algum tipo de informação sobre FLP ou já ter escutado brevemente, alguma vez, alterações de fala relacionadas à FLP/ DVF, porém não sabiam distingui-las. Considerando que todos os ouvintes não tinham experiência na avaliação da hipernasalidade, supôs-se que eles seriam beneficiados pelo uso de escala com menor variabilidade de graus para classificar a hipernasalidade de fala, fato confirmado pela presente investigação.

Em estudo prévio^(22)^ foi encontrado um percentual de 62,5% de respostas corretas em relação ao padrão-ouro na classificação da hipernasalidade em amostras de fala contendo apenas sons orais (alta pressão), realizadas por fonoaudiólogas sem experiência, utilizando a escala de 4 pontos^(22)^. Ao comparar esse percentual com os achados do presente estudo, observa-se um valor inferior para a escala de 4 pontos e um valor superior para a escala de 3 pontos. Isso sugere que, para alunos sem qualquer vivência ou experiência clínica, a escala reduzida foi favorável, enquanto a escala de 4 pontos dificultou as análises realizadas.

Em estudo anterior^(24)^, no qual a classificação da hipernasalidade foi realizada por alunos das séries iniciais de um curso de Fonoaudiologia, foi obtido um percentual de 66% de acertos em relação à avaliação padrão-ouro para as análises de amostras orais de baixa pressão, utilizando a escala de 4 pontos. No presente estudo, um percentual inferior foi obtido para amostras de alta pressão nas análises realizadas pelos alunos, também utilizando a escala de 4 pontos. A inclusão de amostras constituídas por um conjunto de 12 frases de alta pressão^(9)^, pode ter dificultado a classificação da hipernasalidade pelos alunos, usando a escala de 4 pontos, devido a outras alterações de fala coexistentes, como emissão de ar nasal e/ou articulações compensatórias. Por outro lado, resultados similares ao estudo prévio^(25)^ foram obtidos no presente estudo, utilizando a escala de 3 pontos, sugerindo que esta escala favoreceu as análises quando alterações de fala coexistentes não foram controladas.

Alguns estudos propõem a inclusão de amostras de referência em análises perceptivas realizadas para avaliação das alterações de fala relacionadas à FLP/DVF tanto por fonoaudiólogos sem^(20,22)^ quanto com experiência^(6)^. As referências têm como objetivo favorecer e aumentar a consistência das respostas, uma vez que a instabilidade nos padrões internos dos avaliadores pode ser minimizada por referências perceptivas, promovendo maior concordância entre ouvintes^(6)^. Em estudo prévio^(16)^ envolvendo ouvintes não experientes, estudiosos ofereceram duas amostras de referências (uma com ausência e outra com presença de hipernasalidade grave). Essas amostras foram apresentadas aos ouvintes antes da tarefa de julgamento e repetidas a cada cinco amostras, antes de os ouvintes (crianças) analisarem as amostras usando a escala de três pontos.

Diferentemente do estudo prévio mencionado^(16)^, no presente estudo, não foram disponibilizadas amostras de referência ou treinamento que pudessem interferir nas análises realizadas pelos alunos utilizando as duas escalas propostas (quatro e três pontos). Antes de iniciar as análises, a pesquisadora somente realizou uma breve apresentação em *PowerPoint* para os alunos, abordando definições sobre FLP, DVF e hipernasalidade, com o objetivo de familiarizá-los com os temas, o aspecto de fala a ser avaliado e as escalas. Os procedimentos empregados no presente estudo tiveram como foco verificar exclusivamente o efeito das escalas sobre as análises dos ouvintes não experientes, sem interferências de referências perceptivas.

Este estudo também verificou os índices de concordância Kappa (e sua interpretação) das análises dos alunos em comparação com a avaliação padrão-ouro, utilizando as escalas de quatro e três pontos. Os resultados revelaram uma concordância regular para a escala de quatro pontos e moderada para a escala de três pontos, indicando maior confiabilidade nas análises realizadas com a escala de três pontos. Além disso, o aumento significativo do índice de concordância para a escala de 3 pontos (com base no intervalo de confiança de 95%) em relação ao índice da escala de 4 pontos, como observado neste estudo, sugere que a escala reduzida favoreceu a confiabilidade das análises dos alunos em relação à avaliação padrão-ouro. Por outro lado, os achados do estudo mostram que o uso da escala de 4 pontos por ouvintes não experientes pode dificultar análises perceptivas dos graus da hipernasalidade, o que também foi notado em investigação anterior^(22).^

A concordância moderada encontrada no estudo (escala de três pontos) pode ser explicada pela caracterização dos ouvintes (alunos sem experiência), pelos procedimentos metodológicos empregados (não inclusão de amostras de referência) e pelas amostras de fala incluídas (sem controle de coexistência de outras alterações de fala). De acordo com estudiosos, a hipernasalidade, quando associada às articulações compensatórias (uso de ponto articulatório atípico), pode ser julgada perceptivamente como mais nasalizada^(10)^. Embora as amostras de fala do estudo tenham sido controladas para disfonia devido ao seu possível impacto na classificação da hipernasalidade^(26)^, outros sintomas de fala como a emissão de ar nasal/turbulência também não foram controlados e, portanto, podem ter impactado a concordância das análises dos avaliadores, em relação à avaliação padrão-ouro.

Um dos objetivos deste estudo foi investigar a porcentagem de concordância das análises intra-avaliador por grupo e escala. Os resultados revelaram uma porcentagem média de concordância das análises duplicadas significativamente maior para a escala de 3 pontos (72%) do que para a para escala de 4 pontos (53,75%). As análises realizadas também indicaram que não houve interação significativa entre grupo (ordem de apresentação) e fator (grau da escala) para o percentual das classificações intra-avaliador, sugerindo novamente que a ordem das classificações realizadas utilizando as escalas não influenciou os resultados da concordância intra-avaliador. Observa-se, portanto, que a escala de três pontos favoreceu a concordância das análises intra-avaliadores.

Foram também obtidos os índices de Kappa intra-avaliador (e sua respectiva interpretação) utilizando as duas escalas. A distribuição absoluta e relativa de concordância intra-avaliador, com base na interpretação Kappa, revelou resultados discrepantes para as escalas de quatro e de três pontos. Na escala de 4 pontos, não houve concordância nas respostas de um avaliador (5%). Houve concordância moderada para 15% dos avaliadores, concordância pobre para 25% dos avaliadores, e substancial para outros 25%, com maior percentual de concordância leve (30% dos avaliadores). Em contraste, para a escala de três pontos, não foram registradas respostas não concordantes. Houve concordância perfeita/quase perfeita para 20% dos avaliadores. Além disso, 20% dos avaliadores apresentaram concordância leve ou substancial. Houve um maior percentual de concordância moderada (35%), enquanto a interpretação pobre ocorreu apenas para um avaliador (5%). Conjuntamente, estes achados sugerem uma concordância intra-avaliador mais favorável para a escala de três pontos, indicando uma maior confiabilidade nas análises intra-avaliadores para esta escala.

Discussões sobre procedimentos que podem favorecer a análise perceptivo-auditiva da hipernasalidade de fala são frequentes entre estudiosos^(5,24)^. Alguns expressam reservas em relação à validade de escalas categóricas na análise de características de fala para fins clínicos e de pesquisa^(8,12,13,27)^, argumentando que os ouvintes não percebem intervalos (ou categorias) exatamente iguais durante suas análises^(26)^, tendendo a subdividir o extremo inferior da escala em intervalos menores^(13)^. No entanto, as escalas com intervalos iguais ou ordinais têm sido o recurso mais utilizado por clínicos e por pesquisadores^(9,20,28)^ especialmente na rotina clínica, visto que essas escalas parecem ser intuitivamente de fácil aplicação, permitindo a comparação de achados entre escalas e avaliadores^(14)^.

Os resultados deste estudo indicam que, ao empregar escalas categóricas em análises perceptivas, a escala de 3 pontos pode favorecer a classificação da hipernasalidade por ouvintes sem experiência, concordando com achados anteriores^(16)^. Recomenda-se que a escala reduzida seja inicialmente adotada em vivências clínicas com alunos em formação. O uso de escalas com menos graus ou categorias pode beneficiar a tarefa de resolução nas análises perceptivas.

O presente estudo contribui significativamente para um melhor entendimento da influência do grau de pontuação das escalas em análises perceptivas realizadas por ouvintes não experientes, ou seja, alunos em formação sem conhecimento ou experiência clínica relacionada às alterações de fala na FLP/DVF. Como destacado em estudo prévio^(16)^, escalas categóricas reduzidas podem aprimorar a confiabilidade da classificação da hipernasalidade de ouvintes não treinados. Nesse sentido, é necessário dar continuidade à realização de estudos que empreguem escalas reduzidas para esses ouvintes. Esse argumento é fundamentado nos achados de estudos anteriores que indicaram pouco ou nenhum efeito de treinamentos perceptivos-auditivo breves direcionados à classificação da hipernasalidade de fala, utilizando a escala de 4 pontos, por fonoaudiólogos não treinados^(22)^ ou residentes em otorrinolaringologia^(29)^. Sugere-se que, além de treinamentos estruturados e mais longos, a implementação da escala reduzida, como parte desses treinamentos, pode aprimorar as classificações perceptivas desses ouvintes.

Embora a tarefa experimental do estudo tenha sido supervisionada pela pesquisadora, os alunos utilizaram seus próprios fones de ouvido. A pesquisadora monitorou todos os alunos para garantir o uso do mesmo fone de ouvido nos dois dias de análises, ocorrendo em ambiente sem ruídos. Contudo, em estudos futuros, recomenda-se o uso de fones de ouvidos controlados e a realização das avaliações em um mesmo local. Vale ressaltar que os registros da fala em áudio com equipamentos de qualidade para análise de avaliadores múltiplos e padronização de estímulos de fala foram controlados no estudo, uma vez que são consideradas estratégias importantes para se alcançar confiabilidade de análises perceptivas^(20)^. No entanto, não houve controle das coexistências de alterações nas amostras de fala, o que pode ter impactado na classificação da hipernasalidade em ambas as escalas. Para estudos futuros, sugere-se a comparação da análise entre escalas de graus distintos, considerando o controle de outros sintomas de fala relacionados à FLP/DVF.

## CONCLUSÃO

A escala de três pontos proporcionou uma melhor classificação em relação ao grau da hipernasalidade de fala feita pelos alunos de um curso de Fonoaudiologia. A porcentagem média de respostas corretas em comparação com a avaliação padrão-ouro foi significativamente maior para esta escala quando contrastada com os resultados obtidos com a escala de quatro pontos. A análise da concordância intra-avaliadores revelou diferenças significativas entre as duas escalas, apresentando uma maior porcentagem de respostas intra-avaliadores concordantes para a escala de três pontos. A ordem de apresentação das escalas não influenciou as classificações realizadas. As análises baseadas na estatística Kappa demonstraram uma maior concordância intra-avaliadores nas classificações feitas com a escala de três pontos.
